# Functional subsets of circulating follicular helper T cells in patients with atherosclerosis

**DOI:** 10.14814/phy2.14637

**Published:** 2020-11-23

**Authors:** Atefe Ghamar Talepoor, Shahdad Khosropanah, Mehrnoosh Doroudchi

**Affiliations:** ^1^ Department of Immunology School of Medicine Shiraz University of Medical Sciences Shiraz Iran; ^2^ Department of Cardiology School of Medicine Shiraz University of Medical Sciences Shiraz Iran

**Keywords:** cTfh1, cTfh17, cTfh2, NLR, stenosis

## Abstract

Frequencies of circulating T follicular helper (cTfh) functional subsets vary in autoimmune diseases. We evaluated the frequencies and clinical relevance of functional subsets of cTfhs in patients with different degrees of stenosis. Blood samples were collected from high (≥50%) (*n* = 12) and low (<50%) stenosis (*n* = 12) groups and healthy controls (*n* = 6). Three subsets of cTfh cells including cTfh1 (CXCR3^+^CCR6^‐^), cTfh2 (CXCR3^‐^CCX6^‐^), and cTfh17 (CXCR3^‐^CCR6^+^) were detected by flow cytometry. The frequency of cTfh1 cells was higher in control (*p* = .0006) and low‐stenosis groups (*p* = .005) compared to high‐stenosis group. The percentages of cTfh2 and cTfh17 cells were increased in high‐stenosis compared to low‐stenosis (*p* = .002 and *p* = .007) and control groups (*p* = .0004 and *p* = .0005), respectively. The frequency of cTfh1 cells negatively correlated with cholesterol (*p* = .040; *r* = −.44), C‐reactive protein (CRP) (*p* = .015; *r* = −.68), erythrocyte sedimentation rate (ESR) (*p* = .002; *r* = −.79), neutrophil/lymphocyte ratio (NLR) (*p* = .028; *r* = −.67), and cTfh17 (*p* = .017; *r* = −.7244) in the high‐stenosis group. The percentages of cTfh2 and cTfh17 cells positively correlated with cholesterol (*p* = .025; *r* = .77 and *p* = .033; *r* = .71), CRP (*p* = .030; *r* = .61 and *p* = .020; *r* = .73), ESR (*p* = .027; *r* = .69 and *p* = .029; *r* = .70), NLR (*p* = .004; *r* = .76 and *p* = .005; *r* = .74), and with each other (*p* = .022; *r* = .7382), respectively, in the high‐stenosis group. The increased frequencies of cTfh2 and cTfh17 subsets and their correlation with laboratory parameters in patients with atherosclerosis may suggest their role in promoting the inflammatory response and atherosclerosis progression.

## Graphical abstract

The frequencies of cTfh1 cells decrease in the circulation during transition from low‐degree stenosis to high‐degree stenosis, while the frequencies of cTfh2 and cTfh17 increase. The increase in cTfh2 and cTfh17 was the highest in the group with high stenosis.

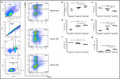

## 1 INTRODUCTION

Atherosclerosis is responsible for several adverse and deadly vascular events such as coronary artery disease (CAD), myocardial infarction, and stroke (Bartlett et al., 2019). Activation or injury of endothelium is suggested to happen early in the course of the events (Mitra et al., 2017). Following the activation of endothelium, expression of adhesion molecules, and secretion of inflammatory cytokines, different immune cells migrate from the circulation into the intima of the arterial wall (Schaftenaar et al., 2016). T cells are found in all stages of the disease in line with macrophages and other leukocytes. The recognition of antigens through major histocompatibility complex (MHC) leads to delivery of effector mechanisms by T cells and contributes in disease development (Ketelhuth & Hansson, 2016). Different subpopulations of CD4^+^ T cells, including Th1, Th2, Th17, Th9, Th22, T regulatory, and T follicular helper (Tfh) cells, are found to accumulate in atherosclerotic lesions (Saigusa et al., 2020a). Despite the thorough investigation of Th1, Th2, and Th17 cells in atherosclerosis, the contribution of other T cell subsets, including Th9, Th22 as well as Tfh cells, in atherosclerosis progression is less investigated (Ahn et al., 2016). The Tfh cells are a CD4^+^CXCR5^+^ T cell subset, essential for the formation of germinal centers (GCs), B cell maturation, and immunoglobulin production in secondary lymphoid organs (Baptista et al., 2018). In addition, a population of Tfh cells called circulatory Tfh (cTfh) cells has been described in the peripheral blood and comprises 15%–25% of memory CD4^+^ T cells in humans (Schmitt et al., 2014). The cTfh cells are composed of heterogeneous populations with different phenotype and distinct functional properties (Ueno, 2016). Based on the expression of CXCR3 and CCR6 chemokine receptors, CD3^+^CD4^+^CXCR5^+^cTfh cells are classified into cTfh1 (CXCR3^+^CCR6^−^), cTfh2 (CXCR3^−^CCR6^−^), and cTfh17 (CXCR3^−^CCR6^+^) cells (Townsend et al., 2010). The cTfh1 cells express the transcription factor T‐bet and produce IFN‐γ. The cTfh2 cells express the GATA3 transcription factor and secrete IL‐4, IL‐5, and IL‐13, while cTfh17 cells express the RORγT transcription factor and produce IL‐17A and IL‐22 (Morita et al., 2011). The cTfh2 and cTfh17 are known as efficient helper cells particularly through secretion of IL‐21 and providing B‐cell help, which increases in many autoimmune diseases (Byford et al., 2018), whereas cTfh1 are defined as nonefficient helper cells and are associated with poor antibody responses (Bentebibel et al., 2013). An alteration in the balance of cTfh1, cTfh2, and cTfh17 cells is associated with the pathogenesis of autoimmune and inflammatory diseases including juvenile dermatomyositis (Morita et al., 2011), adult systemic lupus erythematosus (SLE) (Le Coz et al., 2013), Sjögren's syndrome (Li et al., 2012), multiple sclerosis (Romme Christensen et al., 2013), cancer (Vella et al., 2019), and infectious diseases (Swathirajan et al., 2019). However, little is known about the distribution and function of different cTfh cell subsets in atherosclerosis. In the present study, the frequencies of functional Tfh cell subsets as well as their correlation with clinicopathological parameters were assessed to shed some light on their correlation with the progression of atherosclerosis.

## 2 MATERIAL AND METHODS

### 2.1 Patients

In this case‐control study, blood samples were collected from 12 nonsmoker, nondiabetic participants with 50% or greater stenosis in at least one of the main coronary arteries (six females and six males, mean age ± *SD* =58.41 ± 4.62 years), 12 nonsmoker, nondiabetic individuals with less than 50% stenosis in at least one of the main coronary arteries (six females and six males, mean age ± *SD* =50.83 ± 4.40 years) according to angiography criteria (Maddox et al., 2014), and six nonsmoker, nondiabetic healthy individuals (three females and three males, mean age ± *SD* =48 ± 3.03 years). All subjects with stenosis were selected from individuals referred to hospitals affiliated to Shiraz University of Medical Sciences for diagnostic angiography, and demographic characteristics, clinical, and laboratory data were collected during admission. The exclusion criteria included a positive history of smoking, diabetes, autoimmune diseases, malignancy, inflammatory, or infectious diseases in the last 3 months. A signed informed consent was obtained from all participants. Ethical approval of the study protocol was obtained from the Ethics Committee of Shiraz University of Medical Sciences, Shiraz, Iran. The code of ethical approval of this project was IR.SUMS.REC.1397.1115.

### 2.2 Isolation of peripheral blood mononuclear cells (PBMCS)

Peripheral blood samples were collected separately from all participants. PBMCs were isolated from individuals by density‐gradient centrifugation at 800 × g for 30 min at 25°C using Ficoll‐Paque Plus (GE Healthcare Europe, GmbH, Germany). Freshly isolated PBMCs (1 × 10^6^/ml) were cultured in 10% fetal bovine serum RPMI‐1640 (Shellmax, Iran) overnight at 37°C and were used for further experiments without freezing.

### 2.3 Flow cytometric analysis

PBMCs were stained at 4°C for 20 min with monoclonal fluorochrome‐conjugated antibodies to characterize cTfh cell subsets. The following reagents were used: anti‐CD3‐Alexa Fluor 700 (UCHT1), anti‐CD4‐PerCP (RPA‐T4), anti‐CXCR5‐FITC (J252D4), anti‐CXCR3‐PE/cy7 (G025H7), and anti‐CCR6‐PE (G034E3) from BioLegend (San Diego, CA, USA). Mononuclear cells were separated from peripheral blood and live lymphocytes were identified by forward and side‐angle light scatter characteristics. cTfh cells were identified as CD3^+^CD4^+^CXCR5^+^. Subsequently, cTfh subpopulations were gated from CD4^+^CXCR5^+^ T cells and defined according to CXCR3 and CCR6 expression. Gating was directed to isolating CXCR5^+^CXCR3^+^CCR6^‐^ T cells (cTfh1 cells), CXCR5^+^CXCR3^‐^CCR6^‐^ T cells (cTfh2 cells), and CXCR5^+^CXCR3^‐^CCR6^+^ T cells (cTfh17 cells). Of note, CXCR5 is expressed on cTfh cells; CXCR3 is a specific marker for cTfh1 cells and CCR6 is expressed on cTfh17 cells. We used the single stained tubes for each marker as the basis of gating. At least 200,000 events per sample were collected using FACS Aria II (BD Sciences, San Jose, USA), and results were analyzed using FlowJo software (v7.6.2).

### 2.4 Statistical analysis

The data are expressed as the mean and standard deviation and analyzed with SPSS version 18 software. A two‐sided *p* < .05 was considered as statistically significant. Kruskal‐Wallis test was used for comparison of frequencies of CD4^+^CXCR5^+^ T cells, as well as cTfh1, cTfh2, and cTfh17 subsets between more than two groups. The relationship between variables was evaluated using Spearman's rank correlation test.

## 3 RESULTS

### 3.1 Clinical parameters

The demographic and laboratory characteristics of study participants showed significant differences in the level of several components in patients with stenosis ≥ 50% (high stenosis) compared to those with stenosis < 50% (low stenosis) and healthy controls. Accordingly, C‐reactive protein (CRP) (3.9 ± 0.34 versus 1.95 ± 0.53 and 1.72 ± 0.66 mg/L, *p* = .0001), erythrocyte sedimentation rate (ESR) (35 ± 5.2 versus 22.1 ± 5.1 and 16 ± 2.1 mm/h, *p* = .0001), WBCs (10.8 ± 0.42 versus 9.3 ± 0.45 and 7.9 ± 1.2 10^3^/μL, *p* = .0001), neutrophil count (8.5 ± 0.26 versus 6.3 ± 0.41 and 4.9 ± 0.67 10^3^/μL, *p* = .0001), and neutrophil to lymphocyte ratio (NLR) (4.5 ± 0.47 versus 2.38 ± 0.39 and 1.89 ± 0.1, *p* = .0001) were significantly higher in patients with high stenosis compared to other groups, respectively. However, the lymphocyte count in patients with high stenosis (1.8 ± 0.21 10^3^/μL) was significantly lower (*p* = .0002) compared to those with low stenosis (2.7 ± 0.3 10^3^/μL) and healthy controls (2.6 ± 0.43 10^3^/μL).

### 3.2 Subsets of circulating Tfh cells in peripheral blood mononuclear cells

**Figure 1:**
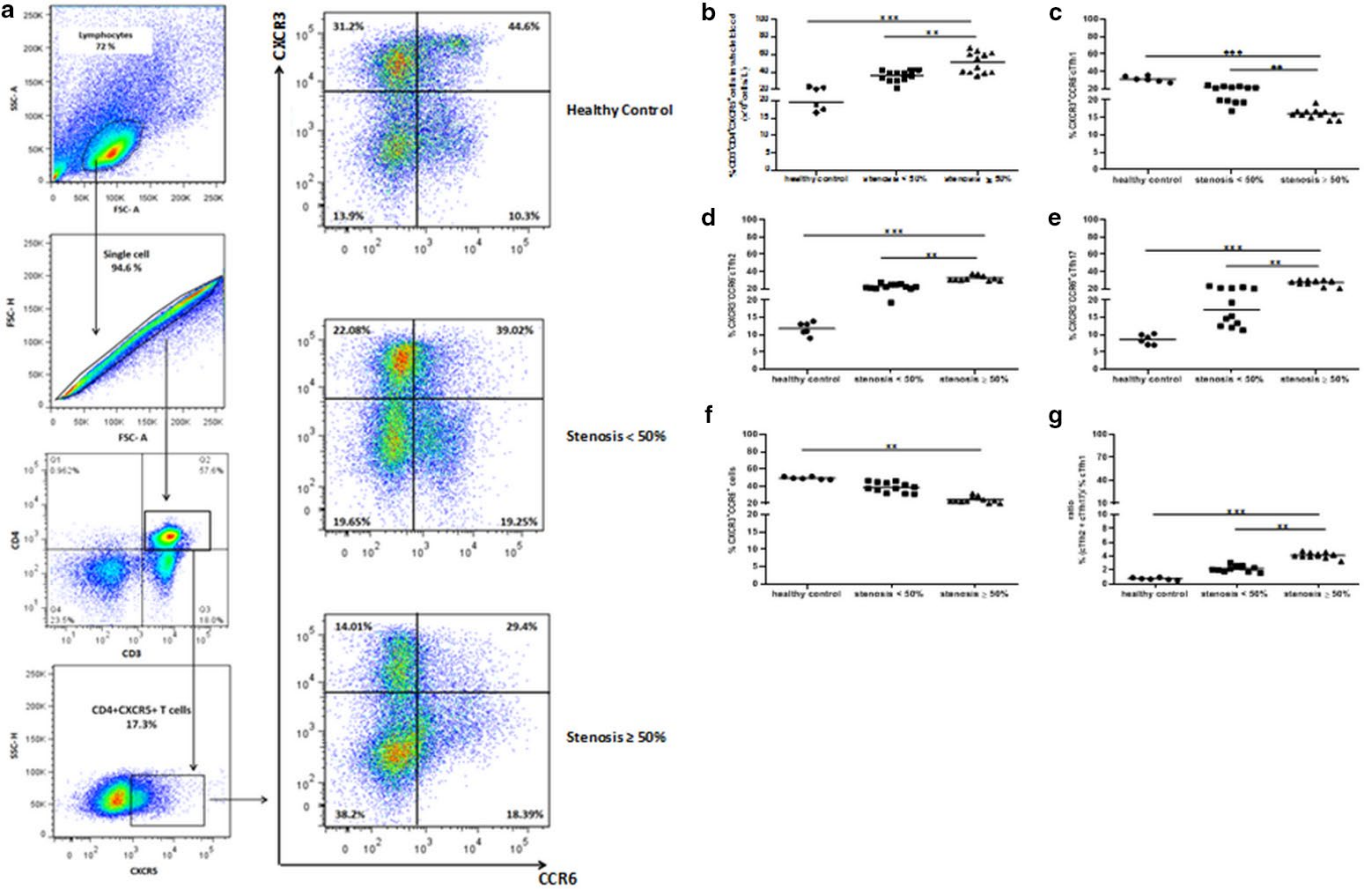
The frequencies of cTfh cell populations were analyzed by flow cytometry. (a) Gating strategy to detect CD3^+^CD4^+^CXCR5^+^T, cTfh1, cTfh2, and cTfh17 in all groups. (b) The frequencies of CD3^+^CD4^+^CXCR5^+^cells, (c) CD3^+^CD4^+^CXCR5^+^CXCR3^+^CCR6^‐^cTfh1 cells, (d) CD3^+^CD4^+^CXCR5^+^CXCR3^‐^CCR6^‐^cTfh2 cells, (e) CD3^+^CD4^+^CXCR5^+^CXCR3^‐^CCR6^+^cTfh17 cells, (f) CD3^+^CD4^+^CXCR5^+^CXCR3^+^CCR6^+^cTfh cells, and (g) ratio of cTfh2 + cTfh17/cTfh1. Each symbol represents an individual; mean are represented by horizontal lines. **p < .01, ***p < .001; Data analysis using the Kruskal‐Wallis test followed by Bonferroni. The results were also evaluated by comparing the raw cell counts and the significance of comparisons stayed valid for all but one analysis with regard to cTfh2 + cTfh17/cTfh1 ratio which became borderline after Bonferroni correction (*p* = .045)

We assessed the frequencies of CD4^+^CXCR5^+^ T cells, CXCR3^+^CCR6^‐^, CXCR3^‐^CCR6^‐^, and CXCR3^‐^CCR6^+^ cTfh subsets in all groups by sequential surface marker gating as shown in Figure 1a. We found that the percentage of CD3^+^CD4^+^CXCR5^+^ T cells was significantly increased in patients with high stenosis compared to individuals with low stenosis and healthy controls (*p* = .007 and *p* = .0009, respectively, Figure 1b). The frequency of CD3 + CD4+CXCR5 + CXR3^+^CCR6^‐^ cTfh1 cells was higher in healthy controls and individuals with low stenosis compared to individuals with high stenosis (*p* = .0006 and *p* = .005, respectively, Figure 1c). The percentage of CD3 + CD4+CXCR5 + CXCR3^‐^CCR6^‐^ cTfh2 cells was significantly increased in patients with high stenosis compared to individuals with low stenosis and healthy controls (*p* = .002 and *p* = .0004, respectively, Figure 1d). As well, the frequency of CD3 + CD4+CXCR5 + CXCR3^‐^CCR6^+^ cTfh17 cells was significantly increased in patients with high stenosis compared to individuals with low stenosis and healthy controls (*p* = .007 and *p* = .0005, respectively, Figure 1e). The frequency of CD3 + CD4+CXCR5 + CXR3^+^CCR6^+^ cTfh cells was higher in the healthy controls compared to individuals with high stenosis (*p* = .008, Figure 1f). The ratio of (cTfh2 + cTfh17)/cTfh1 cells was significantly higher in subjects with high stenosis ≥ 50% than in individuals with low stenosis and healthy controls (*p* = .004 and *p* = .0001, respectively, Figure 1g).

### 3.3 The association of cTfh cell subsets with clinical parameters

**Figure 2:**
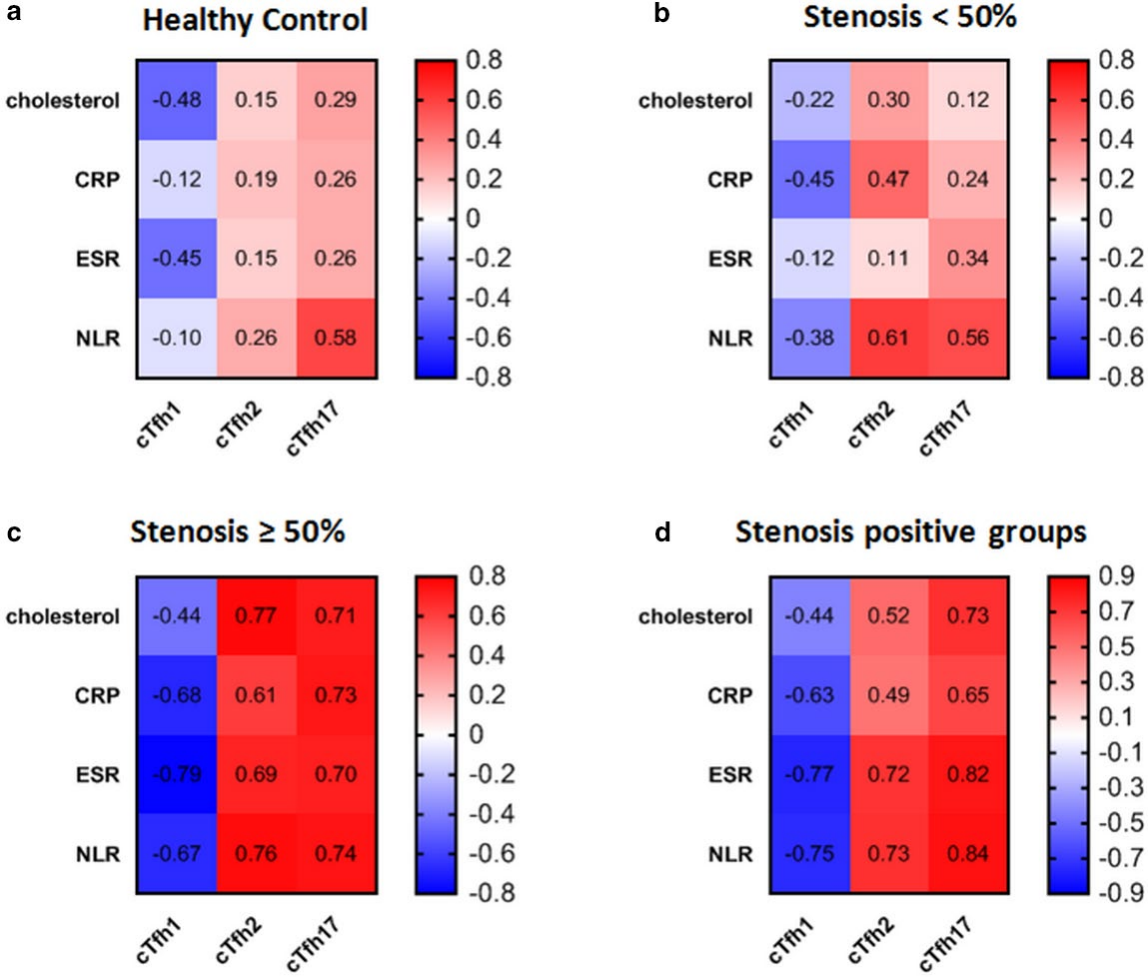
Correlation of laboratoryparameters with the cTfh1, cTfh2, and cTfh17 cells in all study groups. (a) Heatmap correlation of cholesterol, CRP, ESR, and NLR with cTfh1, cTfh2, and cTfh17 cells in healthy controls; (b) Heatmap correlation of cholesterol, CRP, ESR, and NLR with cTfh1, cTfh2, and cTfh17 cells in low‐stenosis group; (c) Heatmap correlation of cholesterol, CRP, ESR, and NLR with cTfh1, cTfh2, and cTfh17 cells in patients with high stenosis and (d) Heatmap correlation of cholesterol, CRP, ESR, and NLR with cTfh1, cTfh2, and cTfh17 cells in stenosis‐positive group (Red: positive correlation, blue: negative correlation). The P‐value and r are determined according to Spearman's rank correlation test

We also investigated the correlation of distinct subsets of cTfh cells and laboratory parameters such as cholesterol, CRP, ESR, and NLR. The results indicated that in the high‐stenosis group, all atherosclerosis‐associated paraclinical parameters negatively correlated with the frequency of cTfh1 and positively correlated with cTfh2 and cTfh17 frequencies in the blood (Table 1, and Figure 2).

**Table 1:** Correlation of laboratory parameters with cTfh cell subsets in all study groups

Parameters	cTfh1		cTfh2		cTfh17	
	*r*	*p*	*r*	*p*	*r*	*p*
Healthy controls						
Cholesterol	−.48	**.029**	.15	.62	.29	.45
CRP	−.12	.87	.19	. 97	.26	.52
ESR	−.45	**.039**	.15	.87	.26	.57
NLR	−.10	.72	.26	.74	.58	**.036**
Stenosis < 50%						
Cholesterol	−.22	.58	.30	.75	.12	.95
CRP	−.45	**.044**	.47	.77	.24	.72
ESR	−.12	.83	.11	.94	.34	.77
NLR	−.38	.63	.61	**.007**	.56	**.038**
Stenosis ≥ 50%						
Cholesterol	−.44	**.040**	.77	**.025**	.71	**.033**
CRP	−.68	**.015**	.61	**.030**	.73	**.020**
ESR	−.79	**.002**	.69	**.027**	.70	**.029**
NLR	−.67	**.028**	.76	**.004**	.74	**.005**
Stenosis‐positive groups						
Cholesterol	−.44	**.039**	.52	**.033**	.73	**.032**
CRP	−.63	**.033**	.49	**.016**	.65	**.004**
ESR	−.77	**.002**	.72	**.019**	.82	**.005**
NLR	−.75	**.004**	.73	**.021**	.84	**.001**

Data shown are “*r* value” and “*p* value”. Bold values show the significant correlations at the .05 level.

CRP, C‐reactive protein; cTfh, circulating follicular helper T cells; ESR, erythrocyte sedimentation rate; NLR, neutrophil/ lymphocyte ratio.

### 3.4 The relationship between different cTfh subsets

**Figure 3:**
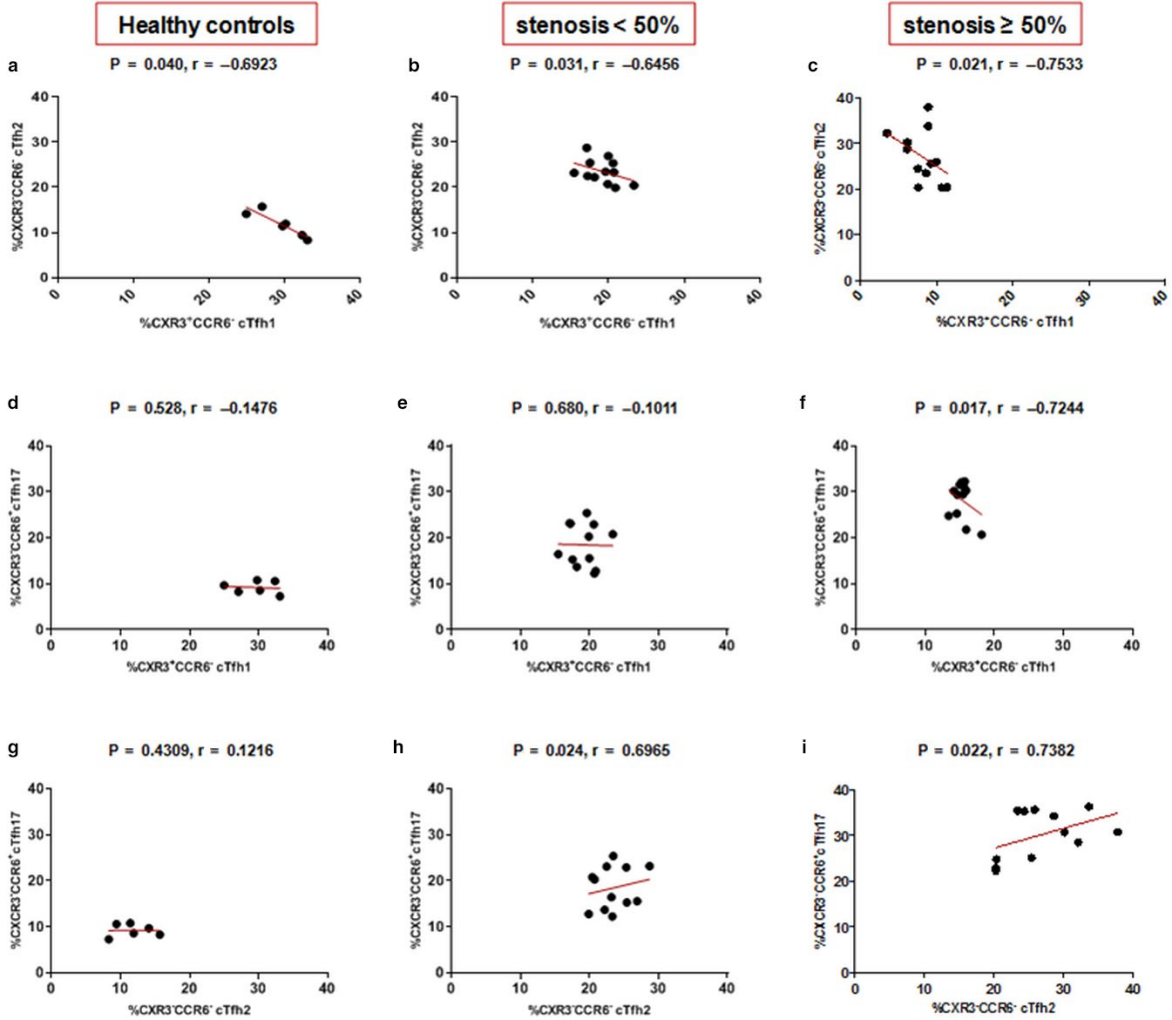
The correlation between cTfh cell subsets in all study groups. (a‐c) Correlation of cTfh1 with cTfh2 cells; (d‐f) Correlation of cTfh1 with cTfh17 cells; and (g‐i) Correlation of cTfh2 and cTfh17 cells.*p* < .05 was considering significant; Spearman's rank correlation coefficient test

We analyzed the correlation between different cTfh subsets in all study groups. We found that while the frequencies of cTfh2 and cTfh17 subsets increased according to the level of stenosis, the frequency of cTfh2 cells inversely correlated with that of cTfh1 in healthy controls (*p* = .040; *r* = −.6923, Figure 3a) as well as in low‐ and high‐stenosis groups (*p* = .031; *r* = −.6456, Figure 3b and *p*= .021; *r* = −.7533, Figure 3c, respectively). The frequency of cTfh17 cells negatively correlated with cTfh1 in high‐stenosis group (*p* = .017; *r* = −.7244, Figure 3f). Conversely, the frequency of cTfh2 cells positively correlated with cTfh17 cells only in low‐ and high‐stenosis groups (*p* = .024; *r* = .6965 Figure 3h and *p* = .022; *r* = .7382, Figure 3i, respectively).

## 4 DISCUSSION

The potential role of T cells as critical drivers and modifiers in the pathogenesis of atherosclerosis has been documented in the past two decades. CD4^+^ T lymphocytes are among the earliest cells to be recruited into the atherosclerotic plaques where they differentiate into diverse subtypes, including Th1, Th2, Th17, and Treg cells, in response to the local milieu of cytokines (Wolf & Ley, 2019). The Th1 and Th17 cells are the most pathogenic T cells in atherosclerosis through secretion of inflammatory cytokines, activation of macrophages, and stimulation of endothelial cells (Wu et al., 2017). Conversely, Treg and Th2 cells play a protective role in atherosclerosis due to the production of anti‐inflammatory cytokines and inhibition of pathogenic T cell responses (Saigusa et al., 2020b). In addition to the previously known CD4^+^ T cell subsets, Tfh cells are most likely proatherogenic by inducing B cell differentiation and antibody response (Gensous et al., 2018). However, there are differences in the function and cytokine secretion profile of Tfh subsets and the role they may play in diseases. In the current study, we observed a lower frequency of cTfh1 cells and higher frequencies of cTfh2 and cTfh17 cells in patients with high stenosis (≥50%) than in healthy controls. The relevance of altered frequency and function of cTfh cells in the progression of different diseases have received considerable attention, recently. Increased frequency of cTfh2 cells is shown to be correlated with the activity of lupus disease (Le Coz et al., 2013). Moreover, increased cTfh17 cells contribute in Sjögren's syndrome where it may be a biomarker of the activation of immune stage (Li et al., 2012). Interestingly, concomitant increase in cTfh2 and cTfh17 cells has been related to the occurrence and development of IgA vasculitis and severe clinical symptoms in patients with Guillain‐Barré syndrome (Xu et al., 2019b). Higher frequencies of CD4^+^IL‐21^+^ T cells are shown to lead to enhanced autoantibody production in SLE patients associated with disease severity (Schmitt et al., 2014). While one could anticipate that all cTfh subsets participate similarly in promoting antibody production, cTfh17 cells, but not cTfh2 cells, secreted IL‐21 in patients with juvenile dermatomyositis (Morita et al., 2011). Further analysis of cTfh cell subsets in our study also revealed a positive association of cTfh2 cells with cTfh17 cells only in groups with positive stenosis. Similarly, the increased frequencies of cTfh2 and cTfh17 cells and reduced frequency of cTfh1 cells are shown in Kawasaki disease (Xu et al., 2019b).

The cTfh1 cells are similar to Th1 cells due to the expression of the transcription factor T‐bet and cytokine IFN‐γ. The cTfh2 cells express the transcription factor GATA‐3 and cytokines IL‐4, IL‐5, and IL‐13, so resemble Th2 cells. As well, cTfh17 cells are similar to Th17 cells because of the expression of the transcription factor RORγT and IL‐17A and IL‐22 cytokines (Ueno et al., 2015). However, the functions of cTfh1, cTfh2, and cTfh17 cells in the pathogenesis of atherosclerosis may not solely be similar to the functions of Th1, Th2, and Th17 cells. Th1 cells play an important role in the development of atherosclerosis by secretion of inflammatory cytokines such as IFN‐γ, IL‐2, TNFα/β and M1 macrophages stimulation (Talepoor et al., 2020). Previous studies have shown that effector (CD4^+^CD45RO^‐^PD‐1^‐^) and memory (CD4^+^CD45RO^+^PD‐1^‐^) Th1 cells secrete large amounts of IFN‐γ and lead to SMC proliferation, collagen production, fibrous cap thinning, and plaque instability (Grönberg et al., 2017; Talepoor et al., 2018). Our results and others’ showed reduced percentage of cTfh1 cells in the high‐stenosis patients compared to the healthy controls (Arroyo‐Villa et al., 2014; Chakera et al., 2012; Che et al., 2016; Xu et al., 2019a). Previous studies have shown that the predominant phenotype of cTfh1 cells is quiescent (ICOS^‐^PD‐1^‐^CCR7^hi^) and only cTfh1 cells with an efficient phenotype (ICOS^+^PD‐1^+^CCR7^lo^) secrete IFN‐γ in a fashion similar to Th1 cells (Schmitt et al., 2014; Ueno et al., 2015). Therefore, it seems that the decrease in cTfh1 cells in high‐stenosis patients is consistent with the decrease in quiescent cTfh cells, which are not able to produce IFN‐γ, IL‐21 as well as antibodies. The proatherogenic or atheroprotective role of Th2 cells in the atherosclerosis process is not fully understood, yet. Studies on Ldlr^‐/‐^ ApoE ^‐/‐^ double knockout mice showed that Th2 cells have an atheroprotective role due to IL‐4 secretion and inhibition of Th1 cells (Fatkhullina et al., 2016; Passos et al., 2020; Talepoor et al., 2020). In contrast, other studies indicated that IL‐4 affected endothelial cells, led to ICAM‐1 expression, inflammatory cytokine and chemokine secretion, caspase‐3 enzyme activation, and endothelial cell apoptosis induction (Ye et al., 2018). Of note, other Th2 cytokines, such as IL‐5 and IL‐13, are atheroprotective through inducing B1 cells to produce natural IgM antibody against ox‐LDL and M2 macrophage induction, respectively (Cardilo‐Reis et al., 2012). Th17 cells by secreting IL‐17, IL‐21, and IL‐22 promote neutrophils, and recruitment of macrophages and atherosclerotic plaque development (Bunte & Beikler, 2019). Conversely, Th17 cells exert atheroprotective role by inhibiting IFN‐γ secretion, increasing collagen production, and stabilizing atherosclerotic plaque (Wu et al., 2017). Therefore, the role of Th2 and Th17 cells in atherosclerosis remains controversial and the effect of these cells on the development of atherosclerosis depends on the stage of the disease. Our results on the increase in cTfh2 and cTfh17 cell frequencies in the high‐stenosis group may suggest different functions of these subsets from that of Th2 and Th17. Collectively, it is logical to assume that the cTfh1 cells with nonefficient helper phenotype and cTfh2 and cTfh17 cells with efficient helper phenotype and IL‐21 secretion may be related to atherosclerosis pathogenesis, probably by subset‐specific cytokine production. Our findings on the negative correlation of cTfh1 cells with cholesterol levels, and positive correlation of cTfh2 and cTfh17 cells with cholesterol levels later in the disease (i.e., patients with stenosis ≥50%) support this notion. Several studies have explored the role of atherogenic dyslipidemia on Tfh cells in mice and human. In this regard, increases in the CXCR3^+^CCR6^‐^ and CXCR3^‐^CCR6^‐^ Tfh cell frequencies are shown to be associated with the elevation of autoantibodies against dsDNA in atherogenic mice along with an increase in the level of IL‐27 (Ryu et al., 2018). Also, IL‐27 was elevated in patients with hypercholesterolemia and was sufficient to induce an increase in Tfh cells (Ryu et al., 2018). Thus, the hyperlipidemia‐IL‐27‐Tfh cell axis might be a probable mechanism in atherosclerosis‐associated SLE in both mice and humans. Moreover, a previous study found that higher level of intracellular cholesterol reduces the expression and signaling of IL‐2R, an inhibitor of Tfh differentiation (Goebel et al., 2005). In this regard, cholesterol attenuates IL‐2 signaling, and induces Bcl6 expression and Tfh differentiation in the context of atherosclerosis (Goebel et al., 2005). Likewise, another study revealed that the 7α, 25‐dihydroxycholesterol and its receptor, the G protein‐coupled receptor EBI2 (GPR183), regulate Tfh cell migration to the proximal B cell zone (Li et al., 2016). Indeed, EBI2**‐**7α, 25‐dihydroxycholesterol interaction promotes Tfh differentiation through quenching IL‐2 signaling (Li et al., 2016). Therefore, it would be interesting to investigate the effect of cholesterol on the differentiation of functional Tfh subsets.

As well, we found that the frequency of cTfh1 cells negatively correlated with CRP and ESR, while the positive correlation between CRP and ESR and frequency of cTfh2 and cTfh17 cells existed in advanced atherosclerosis (patients with stenosis ≥50%). The relationship between high CRP and ESR levels and greater rate of major adverse cardiovascular events is already reported (Bonaventura et al., 2016). In previous studies, it has been shown that both Th17 cells and serum IL‐17 significantly correlated with CRP and ESR values in RA and SLE patients (Bazzazi et al., 2018; He et al., 2020). Bonaventura et al. demonstrated that CRP was positively correlated with the frequencies of neutrophils, total macrophages, and M1 subset in patients with severe atherosclerotic carotid artery stenosis (Bonaventura et al., 2016). A recent study showed that the percentage of cTfh1 cells was negatively correlated with CRP, whereas the percentage of cTfh2 cells was positively correlated with both CRP and ESR in Kawasaki disease (Xu et al., 2019b). Hence, CRP and ESR may contribute to atherosclerosis progression through endothelial NO synthase inhibition, cytokine production, generation of reactive oxygen species by monocytes and neutrophils, vascular stiffness, T cell migration, and activation as suggested in other cardiovascular diseases (Mozos et al., 2017).

Also, we showed a negative correlation between the frequency of cTfh1 and NLR, while the correlation between NLR and the frequencies of cTfh2 and cTfh17 cells were positive in low‐ and high‐stenosis groups. Inflammation is suggested to be a mechanism in the pathogenesis of atherosclerosis and its progression. Neutrophils, by secreting inflammatory mediators, cause vascular wall degeneration while lymphocytes may regulate the inflammatory response and have antiatherosclerotic role (Hoffman et al., 2004). Therefore, NLR has been introduced as an inflammatory biomarker and predictor of cardiovascular disease risk (Venkatraghavan et al., 2015). Accordingly, high NLR was reported to be associated with CAD, acute coronary syndrome, stroke, and composite cardiovascular events (Ikeda et al., 1990; Ommen et al., 1997; Thomson et al., 1980; Zouridakis et al., 2000). Consequently, our findings may signify the cooperation of cTfh2 and cTfh17 cells with neutrophils in atherosclerosis. However, the interpretation of the correlations should be performed with caution, due to the low number of controls in our study. The authors did their best to include as much healthy individuals as possible in a way that it does not undermine the group integrity. The lack of ethical approval for performing angiography on healthy individuals was a restriction on the inclusion of individuals in healthy group.

In conclusion, high frequencies of cTfh2 and cTfh17 subsets in high‐stenosis group and their correlation with cholesterol, CRP, ESR, and NLR suggest an ongoing deviation of nonefficient subsets toward efficient phenotype in the context of inflammation and dyslipidemia and also indicate that these cells, as an effector phenotype, may be involved in atherosclerosis‐related immune responses. To the best of our knowledge, this is the first study to demonstrate the association of circulating cTfh2 and cTfh17 subsets with laboratory parameters of atherosclerosis development in different degrees of stenosis.

## CONFLICT OF INTEREST

The authors declare that they have no competing interests.

## AUTHOR CONTRIBUTIONS

Atefe Ghamar Talepoor: Performed the experiments, analyzed and interpreted the data, and wrote the draft of the paper. Shahdad Khosropanah: Contributed reagents and materials, interpreted the data, and corrected the draft. Mehrnoosh Doroudchi: Conceived and designed the experiments, analyzed and interpreted the data, corrected the draft of the paper, and supervised the research.

## Data Availability

The data that support the findings of this study are available on request from the corresponding author. The data are not publicly available due to privacy or ethical restrictions.
